# The establishment of surrogates and correlates of protection: Useful tools for the licensure of effective influenza vaccines?

**DOI:** 10.1080/21645515.2017.1413518

**Published:** 2018-01-16

**Authors:** Brian J. Ward, Stephane Pillet, Nathalie Charland, Sonia Trepanier, Julie Couillard, Nathalie Landry

**Affiliations:** a Research Institute of the McGill University Health Centre, Infectious Diseases Division, Montreal, QC, Canada; b Medicago Inc, Québec, QC, Canada

**Keywords:** Influenza virus, vaccine, correlates of protection, reference reagents, reference assays, standardization

## Abstract

The search for a test that can predict vaccine efficacy is an important part of any vaccine development program. Although regulators hesitate to acknowledge any test as a true ‘correlate of protection’, there are many precedents for defining ‘surrogate’ assays. Surrogates can be powerful tools for vaccine optimization, licensure, comparisons between products and development of improved products. When such tests achieve ‘reference’ status however, they can inadvertently become barriers to new technologies that do not work the same way as existing vaccines. This is particularly true when these tests are based upon circularly-defined ‘reference’ or, even worse, proprietary reagents. The situation with inactivated influenza vaccines is a good example of this phenomenon. The most frequently used tests to define vaccine-induced immunity are all serologic assays: hemagglutination inhibition (HI), single radial hemolysis (SRH) and microneutralization (MN). The first two, and particularly the HI assay, have achieved reference status and criteria have been established in many jurisdictions for their use in licensing new vaccines and to compare the performance of different vaccines. However, all of these assays are based on biological reagents that are notoriously difficult to standardize and can vary substantially by geography, by chance (i.e. developing reagents in eggs that may not antigenitically match wild-type viruses) and by intention (ie: choosing reagents that yield the most favorable results). This review describes attempts to standardize these assays to improve their performance as surrogates, the dangers of over-reliance on ‘reference’ serologic assays, the ways that manufacturers can exploit the existing regulatory framework to make their products ‘look good’ and the implications of this long-established system for the introduction of novel influenza vaccines.

## Background

One of the ‘holy grails’ of vaccine development is the identification of an easily-standardized and reproducible test that can serve as an accurate predictor of vaccine efficacy across all ages and geographies: a true ‘correlate of protection’.^1,2^ Although a small number of assays come close to this ideal (eg: antibody titres for hepatitis B surface antigen, bacteria toxins like tetanus or the rabies G protein), the best the vaccine community has managed in most cases is the development of ‘surrogate markers’ for immunity and protection. To date, with very few exceptions, all such surrogates have been based upon some aspect of the serologic response (eg: ELISA titres, hemagglutinating or neutralizing antibodies, opsonophagocytic activity, etc.).^3,4^ Pre-formed antibodies are critical to protect against diseases caused by bacterial toxins and can be very helpful in providing immunity against many pathogens and in preventing reinfection with the same or a related pathogen. Despite being sufficient for protection against many infectious challenges, antibodies are critically important for defense against a relatively limited number of micro-organisms: the encapsulated bacteria (*Streptococcus pneumonia*, the meningococci, *Haemophilus influenzae*), the *Enteroviridae* including polioviruses, some enteric bacteria (eg: *Campylobacter* spp.) and *Giardia lamblia*.^5^ Individuals with defects in immunoglobulin production (eg: agammaglobulinemia or hypogammaglobulinemia) can be at considerable risk from these few organisms but generally do not suffer more severe disease with most other pathogens compared to those who are immunologically intact. People with immunoglobulin deficiencies are generally well-protected from the majority of infectious agents by the combined actions of innate immunity and the cellular arm of the adaptive immune response. Why then, has so much effort been placed on defining serologic surrogates of protection in vaccine development programs and in optimizing antibody responses to vaccines?

First, there are many situations in which high titres of pre-formed antibodies provide excellent protection, the most important being the broad immunity provided to the neonate by placentally-transferred maternal antibody.^6,7^ Furthermore, passive immunization in both animal models and human trials has repeatedly demonstrated that many infections can be either prevented entirely or significantly ameliorated by antibodies alone.^8^ Second, the measurement of serum antibodies can be relatively straightforward, inexpensive and rapid. For a large number of infectious diseases, multiple diagnostic platforms with objective readouts are available (eg: immunofluorescence, optical density) that are either fully-automated or are technically simple to perform (eg: enzyme-linked immunosorbent assays or ELISAs). Antigens produced under good laboratory practice (GLP) conditions are often available as are international reference sera so that results can be standardized and reported in international units (IU). For many infections, including most of the vaccine-preventable diseases (VPD), both commercial and ‘in-house’ assays can provide highly reproducible results. These tests are particularly powerful when there is an unchanging target antigen (eg: tetanus or diphtheria toxins) or when the targeted organism is antigenically stable over time and across geography (eg: measles, mumps, varicella viruses). Layers of complexity are added to serologic testing when functional assays are needed to achieve surrogate status (eg; opsonophagocytic antibodies, microneutralization) because of the additional biologic reagents required (eg: cell lines, primary cells, etc.) and because, in many cases, these tests have more subjective read-outs. The value of any given serologic test can also be severely restricted if one or more of the reagents, or the assay methodology itself, is proprietary (eg: pseudovirions for HPV, the ultrasensitive varicella EIA).^9^ To our knowledge, the only non-serologic test that has been accepted by regulatory authorities as full or quasi-surrogate for a VPD is the IFNγ EliSpot that was used to support licensure of the live-attenuated influenza vaccine (FluMist™).^10^ Despite their potential value in vaccine development programs and several decades of intensive effort across multiple disciplines, cellular assays to assess general immune status or responses to infection/vaccination have been difficult to standardize across laboratories.^11-14^

The foregoing paragraphs are the preamble to our contention that influenza vaccines are the ‘poster child’ for how difficult it can be to establish a good serologic correlate of protection (the general characteristics of commercial influenza vaccines are presented in Table 1). Based on the standard classification system that uses the viral surface glycoproteins, hemagglutinin (HA) and neuraminidase (NA), several different influenza virus lineages can infect humans including both seasonal (A, B and rarely C strains) as well as avian viruses (only A strains to date). There is also great genetic diversity of influenza viruses in wild and domestic bird populations as well as several mammalian species that can be infected by these viruses. Furthermore, this entire family of viruses is continuously ‘moving’ in both a piecemeal fashion driven by mutation (ie: genetic ‘drift’) and through wholesale swapping of gene segments between viruses (ie: recombination events or genetic ‘shift’). As a result, each person is exposed to a parade of more-or-less closely-related (ie: seasonal strains) as well as antigenically distinct influenza viruses (ie: pandemic strains) over his or her life-time. The complexity and instability of this host-virus ecosystem requires constant surveillance for drifted and shifted viruses, the annual formulation of influenza vaccines based on educated guess work to predict which strains will circulate in the coming months and repeated vaccination. Each new influenza strain incorporated into a vaccine requires the development of unique reagents (eg: wild-type and reassortant viruses, recombinant proteins, hyper-immune ferret serum) that can be used to assess potency, vaccine-induced serologic responses (ie: HI, MN, SRH and other assays), antigenic relatedness, etc. Some of these reagents are generated by major, international organizations including regulatory agencies (eg: WHO, CDC, NSBIC, FDA) and are made widely available either directly or through reagents ‘depots’ like Biodefense and Emerging Infections Research Resources (BEI Resources: *www.beiresources.org*) while others are generated locally (eg: industry, academic groups). The industry-produced reagents are typically proprietary and are used to evaluate their own vaccine(s). Since these reagents are produced in biological systems as different as eggs and stainless-steel fermenters, there will inevitably be differences between what is ostensibly the ‘same’ reagent produced by company A versus company B as well as between reference and commercial laboratories. Other biological reagents that are required for many of these tests (eg: red blood cells from different animal species, immortalized, cell lines, ferrets) are sourced locally and can also vary widely from year to year and by geography.

**Table 1.** Characteristics of Currently Licensed Influenza Vaccines.

Vaccine Type	Origin	Characteristics and Uses
Live Attenuated (FluMist™)	Embryonated hens' eggs	Quadrivalent (QIV), cold-adapted viruses administered intra-nasally (IN). Available for children/adolescents 2-17 year olds (yo)
Inactivated split-virion (egg-based) (examples: Agrippal™, Fluzone™, Fluvirin™, Fluarix™, FluLaval™, Influvac™)	Embryonated hens' eggs	Trivalent (TIV) or QIV formulations. Viruses chemically-inactivated and detergent split. Administered intramuscularly (IM) or intra-dermally (ID). Varies with the product: available for all ages >6 months (ages 18-64yo for the ID formulation) at 15μg/strain dose. A 60μg/strain high-dose targeted for those ≥65yo (TIV only)
Inactivated split-virion and sub-unit (tissue culture-based) (examples: Flucelvax™, Preflucel™)	Madin Darby Kidney (MDCK) or Vero cells	TIV or QIV formulations. Viruses inactivated and detergent split as above. Partial purification of HA & NA proteins in subunit vaccines. 15μg/strain dose IM. Available for all ages >6 months (and 4-64yo for subunit).
Inactivated split-virion + Adjuvant (FluAd™)	Embryonated hens' eggs	TIV formulation. Viruses inactivated and detergent split as above. MF59 added as an adjuvant. 15μg/strain standard dose IM.
		Targeted for those ≥65yo
Inactivated split-virion + virosomes (Inflexal V™)	Embryonated hens' eggs	TIV inactivated and detergent split as above formulated as ‘virosomes’ with. 15μg/strain standard dose IM. Available for those ≥4yo
Recombinant hemaggulitinin	Baculovirus transfected insect cells (*Spodoptera frugiperda*)	QIV formulation with recombinant HA proteins only. 45μg/strain dose IM. Available for those ≥18yo
(HA) protein (FluBlok™)		

Table 1 illustrates the general types of influenza vaccines licensed in different jurisdictions around the world and their most important characteristics but is not meant to be exhaustive. Not all vaccines are available in all jurisdictions. Some are available as trivalent formulations (TIV: 2xA and 1xB viruses/antigens) while others are available as quadrivalents (QIV: 2xA and 2xB viruses/antigens).

The remainder of this review will discuss i) efforts to standardize the serologic assays routinely used to characterize immune responses to influenza vaccination, ii) limitations of over-reliance on surrogate serologic tests to define immunity to influenza vaccination, iii) potential biases in ‘reference’ reagents and iv) how the current system can act as a barrier to the introduction of novel influenza vaccines. This review is timely because the influenza vaccine ‘landscape’ is rapidly changing and will continue to evolve in the coming years with the introduction of vaccines that differ considerably in their nature (eg: live-attenuated versus inactivated, whole virion versus sub-unit versus recombinant) or their manufacturing technology (eg: embryonated eggs versus mammalian or insect cell culture, plant-based virus-like particles (VLP), etc) from products that have dominated the market for the last half-century.^15,16^

### Standardization of routine influenza serologic assays

As noted above, the serologic assays used to assess protection in adults following routine immunization with inactivated influenza vaccines all use multiple biologic reagents (Table 2). Because of this dependence on intrinsically-variable biologic material, standardization of reagents has been a preoccupation in influenza research for a long time^17^ including national and international efforts to define reference materials (eg: standard sera, consensus virus strains) and to harmonize both reagents and methodologies. Despite such efforts over several decades,^18-21^ the variance in Geometric Mean Titres between industry, academic and public health laboratories performing HI, MN and/or SRH testing on the same samples can still be stubbornly high (eg: 80-fold variation for HI and 109-fold variation for MN in one recent study).^20^ Although comparison to an international standard serum^20^ and standardizing methodologies can improve reproducibility,^21,22^ there is still considerable residual variability in HI testing between laboratories even when both methodologies and reagents are harmonized.^23^ Furthermore, all of these studies have been conducted during single seasons so the reproducibility of testing from year to year even in a single laboratory using the ‘same’ reagents and methods is essentially unknown. Finally, the degree to which any of the biologic reagents on which these assays are based can truly be ‘standardized’ is uncertain. For example, passage of influenza viruses in eggs^24-27^ or cell culture^28^ often introduces mutations that can not only decrease vaccine efficacy^28-30^ but can also seriously confound analytic uses (eg: potency tests based on immune recognition like the single radial immunodiffusion assays (SRID)). Furthermore, both HI and SRH testing use red blood cells obtained from either birds (eg: chicken, turkey) or mammals (eg: horse, guinea pig). Even if the same animal is alive from year to year (eg: a horse vs. chicken), it is not at all certain that RBCs harvested in year 1 will behave the same way in year 2 (E. Montomoli personal communication). It is important to emphasize that these difficulties have nothing to do with the quality of the laboratories themselves which often meet high standards of good laboratory practice (GLP) and are fully-certified by authorities such as Clinical Laboratory Improvement Amendments in the USA (CLIA: https://wwwn.cdc.gov/clia/) or the National Association for Testing Authorities in Australia (NATA: https://www.nata.com.au/nata/) or others.

**Table 2.** Serologic Assays Used to Assess Influenza Vaccine Responses.

Assay	Biologic Reagents Used	Read-Out
Hemagglutination Inhibition Assay (HI)	Source of antigen	Subjective based on degree and timing of RBC agglutination in 96-well plate
	– Either live or whole inactivated virus grown in embryonated hens’ eggs or in tissue culture	
	– Detergent-split antigens from virus grown in embryonated hens’ eggs or in tissue culture	
	– ≥1 recombinant proteins generated in different expression systems	
	– Virus-like particles bearing ≥1 viral protein generated by different platforms	
	Red blood cells (RBC) from different species:	
	– chicken or turkey	
	– horse	
	– guinea pig	
	– human	
	– other	
	Receptor-destroying enzyme (RDE) derived from *Vibrio cholerae* culture supernatant	
Microneutralization Assay (MN)	Live virus	Readouts vary in degree of objectivity (ie: visual inspection of plaques, immunofluorescence, etc)
	– grown in embryonated hens’ eggs	
	– grown in tissue culture	
	Mammalian cell lines	
	– MDCK-II (ATCC CCL-34); MDCK-I; serum free MDCK; MDCK clone CB4; MDCK-Siat cells; LLC-MK2; and HepG2 cells [Meijer 2006]	
Single Radial Hemolysis Assay (SRH)	RBC from different species:	Semi-objective: area of hemolysis typically read by eye using light-box and calipers (note: hemolysis not always symmetrical or clear-cut)
	– chicken or turkey	
	– horse	
	– guinea pig	
	– other	
	Source of Complement	
	– typically rabbit	
	– other species	
	Agarose derived from seaweed	
Enzyme-linked Immunosorbent Assays (ELISA) for IgG, IgG subtypes, IgA, etc.	Source of antigen	Objective: optical density (OD) or immunofluorescence read by machine
	(as per HI assay above)	

### Dominant role played by HI testing

It is worth noting at the outset that all of the ‘standard’ influenza serologies (ie: HI, MN, SRH) primarily measure antibodies directed against the viral hemagglutinin (HA). Among these, the HI test is the most widely used surrogate of protection and several regulatory authorities have established licensure criteria based solely on the HI response [CBER, EMA) as long as there is a commitment to conduct one or more post-licensure efficacy study(ies) (for example^31,32^). A small number of regulators have established similar criteria for SRH data (Japan, EMA) but, to our knowledge, vaccines cannot be licensed in any jurisdiction using SRH data alone. This focus on HI testing is based on early studies that suggested that an HI titre of ∼1:40 was correlated with ∼50% protection against clinical disease in healthy adults,^33-35^ an observation that holds true almost 50 years later, to some extent at least.^36-38^ Indeed, the 1:40 ‘protective cut-off’ value for HI titres has effectively become embedded in the influenza vaccine lexicon/community through long use; achieving near mythical status among vaccine manufacturers as the singular key and least expensive route to licensure (ie: establishing non-inferiority versus a licensed product in terms of the HI response alone). Because of this focus on HI results, industry has single-mindedly pursued the development of vaccines that induce high HI titres. The problems associated with over-reliance on HI testing have recently been reviewed.^3^ While it is true that HI results tend to be well-correlated with other serologic assays (eg:^39^ others) and are generally predictive of protection in healthy young adults, the widely-cited 1:40 cut-off for 50% protection is probably far too low for children. Several recent studies have suggested that HI titres between 1:260-1:320 for different strains may be needed in children to predict a similar level of protection.^39-41^ Even in healthy adults, vaccine failures have been seen with documented titres as high as 1:2048.^42^ HI testing is also far less predictive of protection in the elderly who can derive significant benefit from vaccination despite making little-to-no antibody response (HI, MN, SRH).^43-45^ Although an occasional study suggests that the 1:40 HI cut-off also applies to the elderly when the circulating viruses are well-matched, vaccine-efficacy (VE) can plummet when there is a mismatch.^46^ Furthermore, virtually all of the HI data used for vaccine licensure are based on sera obtained 21-28 days after vaccination despite the fact that HI titres can drop 6-11% per month after vaccination^47^ raising additional questions about the predictive value of this test. Indeed, it is ironic that one of the earliest descriptions of the HI assay as a possible correlate of immunity noted, in the abstract, that subjects with no detectable pre-challenge HI antibodies were better protected than those with low-titres.^33^

Although much harder to measure (reviewed in,^13^) greater cross-protection has recently been reported with antibodies targeting the conserved region of the HA stalk that mediate antibody-dependent cellular cytotoxicity (ADCC).^13,48-50^ Antibodies directed against other targets, most notably neuraminidase (NA), the other influenza virus surface glycoprotein, are also likely to contribute to protection but are rarely measured.^51^ Indeed, recent work with an H1N1 human challenge model suggests that anti-NA titres are more predictive of protection than HI levels.^52^ Finally, cellular responses are critical for recovery from and memory against virtually all viral pathogens and natural influenza infection elicits strong CD4^+^ and CD8^+^ T cell responses.^53-55^ The cellular response is directed against conserved epitopes of both surface and internal viral proteins.^56-59^ Although neither easy nor inexpensive,^13^ T cell assays would very likely be excellent alternate predictors of immunity in influenza.^60^ For example, in mouse models of influenza infection, protection can be achieved by transfer of either CD4^+^ or CD8^+^ T cells alone.^61,62^ Given the pivotal role that CD4^+^ T cells play in supporting both B and CD8 T cell function,^63^ it is not surprising that T_helper_ cells have been proposed as potential correlates of vaccine protection against influenza^64,65^ The induction of specific CD4^+^ T subpopulations (including, but not restricted to, CD4^+^ follicular T cell) by vaccination appears to be a good marker of long-term antibody response.^66,67^ While T cells alone may not provide ‘sterile immunity’ under normal conditions (ie: complete prevention of infection),^68^ it is likely that influenza-specific T cell memory will modulate disease and/or prevent severe outcomes. Indeed, pre-existing influenza-specific CD4^+^ T cells were shown to protect against symptomatic illness in both H3N2 and H1N1 human challenge models^69^ and CD8^+^ T cell responses were correlated with better clinical outcomes during the 2009 H1N1 pandemic in United Kingdom.^70^ Cellular responses may be particularly important in protecting the elderly who tend to have weak HI antibody responses to vaccination.^71-73^ Recent studies have suggested that cellular responses to influenza antigens can be enhanced by some adjuvants (eg: TLR-ligands, ASO3)^74-76^ and/or delivery in particulate form such as virus-like-particles (VLPs),^77^ including those produced by us in plants (eg:^78,79^).

Despite years of effort in the oncology, transplant and HIV research communities, the twin Achille's heels of cellular assays as clinical correlates are their technical difficulty (ie: standardization) and cost (ie: typically orders of magnitude more expensive than serology). Although these assays can be highly reproducible over time in the same laboratory,^80^ inter-laboratory variability, appropriate controls and the automated analysis of high-dimensional data have been major stumbling blocks until recently.^81,82^ Nonetheless, regulatory authorities like the European Medicines Agency and the National Institute for Biological Standards and Control are increasingly interested in a broader evaluation of the immune response, including cellular responses, elicited by influenza vaccines.^83,84^ Such regulatory openness is important since several candidate vaccines currently at different stages of development do not contain any epitopes that would be expected to elicit serum HI antibodies^85^ (reviewed in).^86^ In summary, the recent efforts to better understand vaccine-induced correlates of immunity in influenza strongly suggest that the past half-century spent trying to make vaccines that induce large quantities of HI antibodies may have been, to some extent at least, misguided or at least restrictive.

### Potential confounding and bias in so-called ‘reference’ reagents and ‘standard’ tests

The problems with reagent and assay standardization outlined above would be difficult enough if the hurdles were random. They are not. One of the earliest techniques for isolating and expanding influenza viruses was the use of embryonated hens’ eggs and this time-honoured approach for growing virus has been the foundation of virtually all influenza vaccine production until the recent addition of cell-culture capacity in some countries.^87^ The embryonated egg platform therefore logically became the source for all virologic reagents; reference strains, antigens, purified proteins. At least, this was logical until it was recognized that influenza viruses grown in eggs will inevitably accumulate mutations as they adapt to optimize growth in their new environment^88^ even though such mutations can be favourable in terms of yield.^89^ However, when these mutations occur in locations critical for an effective immune response (eg: the receptor binding site of HA), the negative consequences can be substantial and varied. First and foremost, the purpose of seasonal influenza vaccination is to protect people and not chickens or eggs; so mutations that change important viral targets away from the wild-type have the potential to lower efficacy. Recent evidence suggests that egg-based vaccines do, in fact, induce antibodies that target egg-adapted strains better than wild-type viruses^27^ and that such mutations can decrease VE.^29^ Although viruses grown in mammalian cell culture are theoretically under less mutation pressure, growth in any artificial environment has the potential to drive adaptive mutation.^28,88^ Furthermore, some wild-type viruses can be difficult to isolate or amplify directly in tissue culture so are initially passaged in eggs.^24,90^ Even a single egg passage may be sufficient to introduce one or more immunologically-important mutations that can then persist in subsequent tissue culture. In addition to their potential impact on yield and VE, these adaptive mutations can also affect the properties of egg- and tissue culture-derived viruses as reagents for potency and receptor binding assays, infectivity, serotyping and serologic testing.^88,91-93^ Since almost all ‘reference’ reagents are produced in either eggs or tissue culture, these reagent effects can seriously confound analytic and evaluative work in the development of new vaccines that are not egg- and/or tissue-culture derived (eg: recombinant systems such as Medicago's plant-made virus-like particles^94^ and Protein Sciences baculovirus-expressed HA^95^) or that protect by different mechanism(s)(eg: live-attenuated influenza vaccine: FluMist).^10^ Therefore, an egg-derived vaccine is much more likely than a recombinant (ie: wild-type) vaccine to induce antibodies that react in ‘standard’ serologic testing (eg: HI, MN, SHR) when a ‘reference’ egg-based reagent is used in these assays. Regardless of whether or not these antibodies are clinically useful, an egg-based vaccine will likely have a substantial advantage in any head-to-head serologic comparison with a non-egg-based technology when the reagents used in the assays are derived from egg-adapted viruses. This type of reagent bias was unambiguously demonstrated in clinical trials comparing the immunogenicity and efficacy of egg- or tissue-culture vaccines in both children^90^ and adults.^96^ When the serologic testing was performed with either egg- or tissue-culture-derived reagents there were some striking differences in apparent immunogenicity despite similar demonstrated efficacy. The greatest differences were seen with the B viruses in which egg-adaptation often results in loss of a glycosylation site within the immunologically-critical HA receptor biding domain (RBD: position 196 or 197).^25,97^ Since the shortest and least expensive path to licensure in many jurisdictions is a non-inferiority trial that compares serologic responses of the new vaccine with an existing, licensed vaccine, this kind of reagent bias can have profound impact on the development of novel vaccines (see Examples in box).

**Example 1: FluMist™**

FluMist™ is a live attenuated vaccine delivered intranasally that received FDA approval in 2003 and a ‘preferred’ recommendation from the Advisory Committee on Immunization for children aged 2-8 for 2 years until 2016. The licensure process for this vaccine was far from simple however. Initial clinical studies showed that FluMist did not induce significant serum HI antibody levels and therefore failed to meet the recognized threshold for licensure. However, a large scale trial in over 2000 children showed that >100 IFNγ spot-forming cells per 10^6^ million peripheral blood mononuclear cells was associated with protection; establishing the IFNγ EliSpot test of cell-mediated immunity and not HI antibodies as a better surrogate of protection for this vaccine.^10^

**Example 2: FluBlok™**

FluBlok is a recombinant HA protein vaccine produced in insect cells that received FDA approval in 2013 for adults ≥18 years of age. A recent efficacy study demonstrated a 30% reduction in the risk of PCR-confirmed influenza-like illness for FluBlok compared to a standard dose of inactivated quadrivalent influenza vaccine in adults ≥50 years of age (ie: superior efficacy) despite an apparent inferiority in the induction of HI antibodies for the H1N1 and B/Brisbane strains.^95^ The HA antigens in FluBlok are based on wild-type (WT) sequences but the ‘reference’ reagents used for serologic testing in this study were produced in eggs. These observations highlight the danger of using such ‘reference’ reagents to compare vaccines made using different manufacturing technologies. In this case, the use of egg-derived reagent led to a perception of inferiority yet FluBlok was found to be better at preventing influenza.

**Example 3: Medicago's plant-made VLPs**

**Figure 1.**
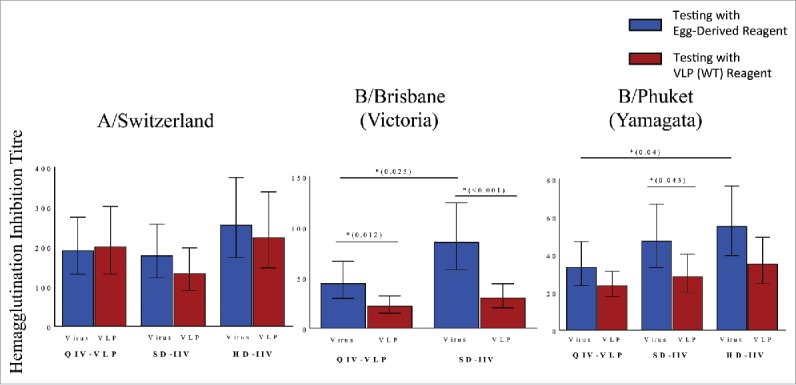
Post-hoc analysis of reagent impact on HAI results in phase II study of a plant-made virus-like particle (VLP) vaccine in elderly subjects ≥65 years of age. Subjects received a single intramuscular dose of a quadrivalent VLP vaccine (QIV-VLP) containing 30mg of each hemagglutinin (HA) used in the 2015-16 seasonal vaccine, a standard dose quadrivalent inactivated vaccine (SD-IIV) containing 30mg of each HA or a high-dose trivalent IIV (HD-IIV) containing 60mg of each HA. Geometric mean titres (GMT) of serum hemagglutination Inhibition (HI) titres were measured in samples from 50 subjects in each arm of the trial using either VLPs (wild-type HA sequence) or reference (egg-derived) reagents in the assay.

The plant-made VLP vaccines produced by Medcago Inc are also based on WT HA sequences but are produced by transient transfection of plants (*Nicotiana benthamiana*).^94^ Both seasonal and pandemic (eg: H5, H7) candidates have shown promise in pre-clinical studies and a candidate quadrivalent VLP vaccine is rapidly advancing through clinical testing. Despite excellent antibody responses to A strain viruses and surprisingly strong CD4^+^ T cell responses to all strains in phase 2 studies, the serologic responses (ie: HI, MN, SRH) to B strain viruses generated by this plant-made vaccine were relatively low compared to 2 different egg-based, inactivated comparator vaccines [^79^, unpublished data]. Since egg-adaptation in most B viruses includes loss of an N-linked glycosylation site in the receptor binding domain (RBD), we wondered if the use of egg-based reagents had contributed to an unanticipated bias in favour of the egg-based vaccines. Characteristics of the four HA proteins of the egg-adapted strains initially used for serologic testing are presented on Table 3 and, indeed, the B virus antigens were either partially or completely lacking an N-glycan in the RBD compared to the WT and VLP proteins. When sera from a subset of 50 subjects in each of the phase 2 studies were re-assessed *post hoc* using either VLPs or WT-like viruses as antigens, HI responses were essentially unchanged for the A viruses but fell significantly for the B viruses in both comparator arms such that differences between the VLP and comparator vaccines observed in the first analysis largely disappeared (Fig. 1). These data strongly suggest that the initial differences in HI titres to the B viruses were an artifact introduced by the use of egg-based reagents in testing. The presence or absence of a carbohydrate (or any other significant amino acid or post-translational change) in this highly immunogenic region of the HA could lead to important differences in the apparent strength (e.g. titer) and/or the repertoire (e.g. cross-reactivity) of the antibody response induced by vaccination. In this context, the use of a ‘standard’ (egg-based) reagent to measure HI titers induced by any vaccine based on WT HA antigens might systematically under-estimate the immunogenicity of a non-egg-based vaccine. Furthermore, the use of egg-derived viruses for both vaccines and ‘reference’ reagents may lead to the false perception of response and protection. In the case of native B viruses for example, RBD epitopes would likely be masked or modified by glycans but antibodies induced by an egg-based vaccine could only be directed against ‘naked’ epitopes.

**Table 3.** Virus Reagents for HI assay (CP-Q14VLP-009 and CP-Q14VLP-010) in Clinical Trials.^*^

Selected Virus Reagents	Amino acid mutation compared to VLP sequence	Changes in N-glycosylation at RBD
A/California/7/2009 (H1N1)-like A/Brisbane/10/2010	6	None
(cell derived)		
NIBSC No. 11/134		
A/Switzerland/9715293/2013 (H3N2)-like A/South Australia/55/2014	1	None
(cell derived)		
NIBSC No. 15/104		
B/Brisbane/60/2008 (NYMCBX-35)	1	Loss of glycoyslation site
(egg-derived)		
NIBSC No. 10/106		
B/Phuket/3073/2013-like	None	∼ 50 % proportion of aglycosylated amino acid when evaluated by MS
B/Utah/9/2014		
(cell-derived)		
NIBSC No. 11/134		

Table 3 illustrates the differences in HA proteins in the context of the available virus reagents selected for the HAI assay in Phase II studies that included comparator vaccines (Note: in those studies, the strains covered by the VLP vaccine were those recommended by WHO for the 2015-2016 Influenza season).

* NCTs 02768805 and 02831751

### The current system as a barrier to novel influenza vaccines

The current regulatory environment for influenza vaccines is an example of how good intentions can occasionally have bad outcomes. The two most common pathways to licensure are i) classical field efficacy studies (versus placebo or an active comparator depending upon the age targeted and the jurisdiction where the studies will be performed) or ii) non-inferiority studies against a licensed comparator. The former tend to be large (9000–30,000 subjects in recent trials)^95,98^ and expensive. They also entail considerable risk since even a large and well-designed study can ‘fail’ if the influenza season is very mild where the study is being conducted, if there is a major mismatch (as occurred in 2012–201:3^29^ or a dominant new virus emerges (eg: as occurred in 2009–2010).^99^ The latter pathway is therefore much preferred - fewer subjects, less expensive and minimal risk as long as the new vaccine has been designed to optimize HA responses.^31,32^ This last caveat constitutes a significant barrier for any new vaccine that relies on other arms of the immune response to provide protection. As highlighted by the examples above, over-reliance on HI results, the egg-based bias of most available ‘reference’ reagents and difficulties in gaining regulatory acceptance of non-serologic tests as predictors of vaccine success are all significant scientific and financial hurdles for novel vaccines. The fact that almost all of the currently licensed vaccines are very similar (egg-based attenuated or split virus at various doses ± adjuvants) is not because this approach works brilliantly. Rather, it is an artifact of the regulatory environment through which these products have to pass. Even the most recently-licensed vaccine (Protein Sciences recombinant HA-based formulation) was almost certainly ‘optimized’ for the induction of HI responses since this formulation contains three times the amount of HA present in most commercial egg-based vaccines.^95^

## Conclusions

Some of the difficulties currently confronting the influenza vaccine community were foreshadowed to some extent by Hobson's iconic 1972 article in which an HI-based correlate of protection was proposed but which also noted that subjects without any detectable HI response were better protected than those with low HI titres.^33^ Given what we have learned about influenza and host-virus interactions over the last half-century, it is somewhat disappointing but not particularly surprising that vaccines optimized for an egg-biased HI response do not perform very well against a family of viruses as varied and as mutationally ‘slippery’ as influenza. It now seems clear that forcing all candidate vaccines to leap through the same serologic ‘hoop’ is probably a mistake since a single surrogate or correlate of protection is highly unlikely to apply to vaccines that work by distinct mechanisms. Despite the cost and difficulties in standardizing the measurement of other aspects of the immune response (eg: ADCC, anti-stalk, CD4^+^ and CD8^+^ T cell responses), it is now almost certain that the best vaccine-induced protection (ie: cross-protective, durable) will be achieved by well-balanced activation of several arms of the immune response rather that a narrow focus on the induction of high HI titres.^2^ In the age of systems biology and a growing awareness of the complexity of the immune system - it is anachronistic to consider only a single element in the immune response in evaluating vaccine-induced immunity.^4,100,101^ In summary, greater effort is needed to investigate and standardize non-serologic assays as possible surrogates/correlates of protection and consideration must be given to ‘vaccine-specific’ rather than ‘universal’ surrogates/correlates of protection. Finally, the influenza vaccine community needs to be vigilant to ensure that tradition, ‘reference’ assays and ‘reference’ reagents do not become barriers to the introduction of novel and potentially more effective vaccines.
